# Astrocytes and microglia in neurodegenerative diseases: Lessons from human *in vitro* models

**DOI:** 10.1016/j.pneurobio.2020.101973

**Published:** 2021-05

**Authors:** Hannah Franklin, Benjamin E. Clarke, Rickie Patani

**Affiliations:** a The Francis Crick Institute, 1 Midland Road, London, NW1 1AT, UK; b Department of Neuromuscular Diseases, UCL Queen Square Institute of Neurology, Queen Square, London, UK

**Keywords:** Aβ, Amyloid-β, AD, Alzheimer’s disease, APOE, Apolipoprotein E, APP, Amyloid precursor protein, BMPs, Bone morphogenetic proteins, ALS, Amyotrophic Lateral Sclerosis, C9ORF72, Chromosome 9 open reading frame 72, CCL2, Chemokine (C-C motif) ligand 2, CNS, central nervous system, CNTF, Ciliary neurotrophic factor, EGF, Epidermal growth factor, FACS, fluorescence activated cell sorting, FGF, Fibroblast growth factor, FTD, Frontotemporal dementia, GBA1, Glucocerebrosidase beta enzyme 1, GD, Gaucher disease, HD, Huntington’s disease, HTT, Huntingtin, IFN-γ, Interferon gamma, GM-CSF, Granulocyte-macrophage colony-stimulating factor, hiPSCs, human induced pluripotent stem cells, JAK-STAT, Janus kinase-signal transducer and activator of transcription, LIF, Leukemia inhibitory factor, LPS, lipopolysaccharide, LRRK2, Leucine-rich repeat kinase 2, M-CSF, Macrophage colony stimulating factor, NFIA, Nuclear factor 1 A-type, NGF-β, Nerve growth factor, NPCs, neural progenitor cells, PD, Parkinson’s disease, sALS, sporadic ALS, SOD1, Superoxide dismutase 1, SOX9, SRY-box 9, TDP-43, TAR DNA-binding protein 43, TGF-β, Transforming growth factor beta, TNF-α, Tumor necrosis factor alpha, TREM2, Triggering receptor expressed on myeloid cells 2, VCP, Valosin-containing protein, Astrocyte, Microglia, Neurodegenerative disease, Human iPSC, *In vitro*

## Abstract

Both astrocytes and microglia fulfil homeostatic and immune functions in the healthy CNS. Dysfunction of these cell types have been implicated in the pathomechanisms of several neurodegenerative diseases. Understanding the cellular autonomy and early pathological changes in these cell types may inform drug screening and therapy development. While animal models and post-mortem tissue have been invaluable in understanding disease processes, the advent of human *in vitro* models provides a unique insight into disease biology as a manipulable model system obtained directly from patients. Here, we discuss the different human *in vitro* models of astrocytes and microglia and outline the phenotypes that have been recapitulated in these systems.

## Highlights

• Astrocytes and microglia key fulfil homeostatic and immune functions in the CNS.
• Dysfunction of these cell types is implicated in neurodegenerative diseases.
• Understanding cellular autonomy and early pathogenic changes is a key goal.
• New human iPSC models will inform on disease mechanisms and therapy development.

## 1 Introduction

Astrocytes and microglia possess crucial homeostatic roles that are vital for neuronal function and survival, including metabolic support and synaptic regulation (Salter and Stevens, 2017; Vasile et al., 2017). Together, both cell types comprise the main immune component of the intact central nervous system (CNS) and can undergo dramatic reactive changes in response to their environment, with subsequent beneficial or detrimental effects on surrounding neurons (Anderson et al., 2016; Liddelow et al., 2017; Butovsky and Weiner, 2018). However, the spatiotemporal interplay between these cell types and their reactive states is only beginning to be resolved. Dysfunction of homeostatic processes and transformation to a reactive state in both astrocytes and microglia has been implicated in the pathomechanisms of several neurodegenerative diseases (Philips and Rothstein, 2014; Karran and De Strooper, 2016; Hickman et al., 2018; Clarke and Patani, 2020).

Although astrocytes and microglia share some common functions, their developmental origin is vastly different. Like neurons, astrocytes derive from the neuroectoderm and their precursors undergo a temporally regulated and likely epigenetically mediated gliogenic switch dependent on cardiotrophin and Notch signalling (Sloan and Barres, 2014; Tchieu et al., 2019). This process occurs through the activation of the Janus kinase-signal transducer and activator of transcription proteins (JAK-STAT) pathway and the critical transcription factors, SRY-box 9 (SOX9) and Nuclear factor 1 A-type (NFIA) (Kamakura et al., 2004; Kang et al., 2012). Astrocyte precursors migrate radially from the ventricular zone where they terminally differentiate and occupy mostly non-overlapping domains (Ge et al., 2012; Tien et al., 2012; Tsai et al., 2012). Both region specific encoding and local microenvironmental cues consolidate astrocytic identity likely depending on the specific requirements of interacting neurons within a particular neuroglial unit (Clarke et al., 2020a). Once mature, few astrocytes are proliferative even in response to either injury or disease (Colodner et al., 2005; Sofroniew and Vinters, 2010; Liddelow and Barres, 2017).

Meanwhile, microglia arise from mesodermal cells in the yolk sac which invade the CNS early during development, preceding astrogliogenesis (Ginhoux et al., 2010; Gomez Perdiguero et al., 2015). These yolk sac derived erythro-myeloid progenitors establish a distinct microglial identity different to macrophages through a combination of both ontological and environmental cues (Gosselin et al., 2017; Bennett et al., 2018). Microglial differentiation is independent of the transcription factor MYB but reliant on two other transcription factors, PU.1 and IRF8 (Schulz et al., 2012; Kierdorf et al., 2013). Consolidation of microglial identity is dependent on Macrophage colony-stimulating factor (M-CSF), Transforming growth factor beta (TGF-β) and IL-34 signaling (Butovsky et al., 2014; Muffat et al., 2016; Abud et al., 2017; Amos et al., 2017; Utz et al., 2020). Once mature, microglia establish non-overlapping domains in the adult CNS and, unlike astrocytes, remain highly mobile (Davalos et al., 2005; Nimmerjahn et al., 2005). The microglial pool is maintained locally without peripheral myeloid contribution and can proliferate upon stimulation with certain reactive stimuli (Bruttger et al., 2015; Tay et al., 2017; Rossi et al., 2018).

In neurodegeneration research, poor translation of findings from preclinical models has been in part attributed to the failure of animal models to faithfully recapitulate disease phenotypes. Human astrocytes and microglia display differences to their mouse counterparts in terms of their morphology, gene expression and function (Oberheim et al., 2012; Tarassishin et al., 2014; Zhang et al., 2016; Galatro et al., 2017; Gosselin et al., 2017). Access to post-mortem human astrocytes or microglia is limited and only provides information of the end stage of the disease. The seminal finding of the reprogramming of somatic nucleated mouse (Takahashi and Yamanaka, 2006) and later human cells (Takahashi et al., 2007) into induced pluripotent stem cells (hiPSCs) has greatly enhanced the ability of researchers to study different human CNS cell types *in vitro*, including both astrocytes and microglia. These advancements have enabled the investigation of the primacy of pathological events, cellular autonomy and cell type specific pathobiology in a human model while opening new possibilities for drug screening and development. hiPSC derived astrocytes and microglia allow for the study of highly pure populations of these cell types directly from patients, expressing mutations at physiological levels. Furthermore, the use of zinc finger nucleases or CRISPR technology has enabled the attainment of isogenic control lines, insertion of disease-causing mutations or knockout of disease-relevant genes, enabling the study of specific disease-causing genes in cell biology. However, it is important to consider the limitations of any model system, as each has advantages and disadvantages depending on the scientific question being addressed. Human *in vitro* models are more time-consuming and expensive to make than other *in vitro* models and cannot truly model the *in vivo* environment. It is also more difficult to model the complex multiple cell-cell interactions that exist *in vivo*. Furthermore, hiPSCs represent a fetal maturational status which do not capture some age-related phenotypes that are relevant to the study of neurodegenerative diseases (Patani et al., 2012; Ziff and Patani, 2019) and in particular to the study of astrocytes and microglia, which undergo major transcriptional changes during aging (Soreq et al., 2017). Human *in vitro* models are nevertheless emerging as an indispensable tool for investigating the primacy of pathological events and resolving cell autonomous pathological mechanisms. Here, we summarise the different human *in vitro* models of astrocytes and microglia and discuss recent findings of key disease phenotypes in these models in the context of neurodegenerative disorders.

### 1.1 Human *in vitro* astrocyte models

Protocols for obtaining human astrocytes or microglia *in vitro* attempt to recapitulate steps that occur during development (Table 1) (Tyzack et al., 2016; Haenseler and Rajendran, 2019). Human astrocytes have been derived from a variety of sources including hiPSCs (Krencik et al., 2011; Hall et al., 2017), embryonic stem cells (Gupta et al., 2012), immortalised cell lines (Price et al., 1999; Langley et al., 2009; Furihata et al., 2016), directly reprogrammed from fibroblasts (Meyer et al., 2014) or immunopanned from fetal or adult healthy donors (Zhang et al., 2016), each with their own advantages and disadvantages (Table 2). Human *in vitro* astrocyte models are able to recapitulate several physiological functions including glutamate uptake, inflammatory and calcium responses to stimuli and promoting neuronal synapse formation, neurite outgrowth and electrophysiological maturation (Hall et al., 2017; Bradley et al., 2019; Taga et al., 2019; Thelin et al., 2020). Furthermore, human astrocyte progenitors can be implanted into immunosuppressed rodents where they later express mature astrocyte markers and promote improved performance in cognitive tasks (Han et al., 2013; Haidet-Phillips et al., 2015).

**Table 1:** Summary of different human *in vitro* astrocyte protocols.

Citation Reference	Summary
Krencik et al. (2011)	Immature astrocytes specifiable to the forebrain or spinal cord with FGF8 or RA, respectively, from hiPSCs using a 180-day protocol. Progenitors expanded with EGF + FGF2 for >150 days and then terminally differentiated with CNTF for 7 days. Astrocytes elicited electrophysiological responses to glutamate, propagated calcium waves upon mechanical stimulation, performed glutamate uptake and promoted synapse formation of co-cultured neurons.
Gupta et al. (2012)	Human ES cell derived astrocytes obtained through a combination of BMP-mediated Smad and LIF-mediated JAK-STAT signalling. Neuroprotective properties of astrocyte conditioned media after exposure of human ES cell derived neurons to oxidative stress through glutathione-dependent and independent mechanisms.
Serio et al. (2013)	Astrocytes from *TARDBP* mutant hiPSCs. NPCs grown in suspension as neurospheres, enriched with LIF and EGF (4−6wks) followed by expansion with EGF and FGF2 and then CNTF for terminal differentiation.
Meyer et al. (2014)	Fibroblast derived astrocytes from *SOD1*^A4V^ fALS, *C9ORF72* fALS and sporadic ALS patients. Conversion to tripotent iNPCs through infection with Sox2, KLF4, Oct3/4, c-Myc, followed by switch to medium containing FGF2, EGF and heparin. Astrocytic differentiation initiated through seeding in NPC medium in fibronectin-coated dish, followed by 10% FBS and 0.3% N2.
Zhang et al. (2016)	Purification of astrocytes from fetal, juvenile and adult brains *via* immunopanning technique using anti-HepaCAM antibodies.
Hall et al. (2017)	iPSCs from VCP mutant fibroblasts. Astrocytes generated in monoculture throughout - FGF used to expand and BMP4 and LIF used to terminally differentiate.
Sloan et al. (2017)	Patterning 3D brain spheroids from hiPSCs to dorsal or ventral forebrain fate for up to 590 days. Astrocytes isolated by immunopanning with anti-HepaCAM antibodies perform phagocytic function, promote synapse formation and calcium signalling of co-cultured neurons.
Li et al. (2018)	Expression of NFIA and SOX9 speeds up iPSC derived astrocyte generation which display functional attributes including promoting neurite outgrowth, calcium waves after mechanical stimulation and glutamate uptake.

**Table 2:** Advantages and disadvantages of human *in vitro* astrocyte models.

Model	Strengths	Limitations
hiPSC	High purity	Expensive
	Ability to self-renew	Time consuming
		Developmental model
ESCs	Same as hiPSCs	Same as hiPSCs plus ethical concerns
Transdifferentiated fibroblasts	Preserved age of donor	Limited supply
	Faster than stem cell-based protocols	Reliant on expression of known factors
		Currently reliant on serum affecting reactivity
Immunopanned primary astrocytes	Ability to study cells exposed to *in vivo* cell-cell interactions	Limited supply
	Can obtain from adult donors	
Immortalised cell line	Fast	Karyotype abnormalities
	Ability to self-renew	Abnormal proliferative state
		Currently reliant on serum affecting reactivity

Directed differentiation of astrocytes from hiPSCs generally follows a method of converting hiPSCs to neural progenitors, patterning to a certain region of the neuraxis, propagation *in vitro* for a protracted period of time (*e.g.* 50–70 days) to permit the gliogenic switch followed by terminal differentiation to astrocytes (Tyzack et al., 2016). hiPSCs have the capacity to generate a high purity of terminally differentiated astrocytes from a potentially unlimited population of cells. They also have the advantage over embryonic stem cells of avoiding some ethical considerations. However, deriving astrocytes from this method is time-consuming and costly. Furthermore, hiPSC derived astrocytes are only partially mature compared to adult astrocytes found *in vivo* and are currently unable to capture age-related phenotypes that have been described in astrocytes transdifferentiated from patient fibroblasts (Meyer et al., 2014). Often a combination of 3D embryoid body and monolayer components are used to derive astrocytes, but protocols using exclusively monolayer cultures have also been developed (Hall et al., 2017), allowing for increased enrichment of cultures and therefore reduction of heterologous cell-cell interactions. However, a limitation of monoculture is that it does not mimic the 3D architecture of the developing nervous system. Indeed, the presence of other cell types can be beneficial as cell-cell interactions can be studied and purified populations can be dissociated and sorted (Krencik et al., 2017; Sloan et al., 2017). Many protocols have successfully generated highly pure cultures of mature astrocytes (>90 %) in the absence of fluorescence activated cell sorting (FACS), however FACS can be used as a tool to select for certain astrocyte-specific markers (Barbar et al., 2020).

hiPSCs are typically converted to neural progenitor cells *via* dual SMAD inhibition under feeder-free conditions. Dual SMAD inhibition results in the loss of pluripotency markers such as OCT4 and NANOG and the acquisition of neural progenitor markers such as NESTIN, MUSASHI, PAX6 and SOX1. Regional identity of neural progenitors can be achieved through the application of developmentally-inspired extrinsic cues including diffusible morphogens, such as Wnts, Bone morphogenetic proteins (BMPs), sonic hedgehog along the dorsoventral axis and retinoic acid, Wnts and Fibroblast growth factor (FGF) along the rostrocaudal axis (Krencik et al., 2011; Imaizumi et al., 2015; Hall et al., 2017; Bradley et al., 2019; Clarke et al., 2020b). Neural progenitor cells (NPCs) can be expanded using growth factors such as Epidermal growth factor (EGF) and/or FGF. Over time, neural progenitors spontaneously undergo a gliogenic switch due to the inhibition of neurogenesis and activation of gliogenic JAK-STAT pathways. This process can be accelerated using inducible expression of NFIA and/or SOX9 (Li et al., 2018; Tchieu et al., 2019). Terminal differentiation can be accelerated using BMPs in combination with IL-6 family cytokines, including Ciliary neurotrophic factor (CNTF) or Leukemia inhibitory factor (LIF), through neuregulin application or by exposure to serum. However, serum has been shown to induce a reactive state in astrocytes (Foo et al., 2011; Perriot et al., 2018) and so serum-free approaches to human astrocyte derivation are preferable. In addition to the use of serum, matrix topography has been implicated in driving astrocyte reactivity *in vitro* (Puschmann et al., 2013; Watson et al., 2017). For example, astrocytes cultured in 3D collagen hydrogels and nanofibres are less likely to exhibit reactive phenotypes than those grown in 2D matrices (East et al., 2009; Tiryaki et al., 2015).

As a model of ageing, transdifferentiation of human fibroblasts has the advantage over hiPSC cultures since it has been demonstrated that differentiated neurons retain age related transcripts and display impaired nucleocytoplasmic compartmentalisation due to loss of RANBP17 (Mertens et al., 2015). This model may be particularly useful in the study of neurodegenerative diseases where ageing is a key risk factor. Astrocytes can also be obtained faster than hiPSC-based protocols. However, this method generates a limited supply of cells and currently lacks the ability to pattern neural progenitors to different areas of the neuraxis. Furthermore, published protocols for transdifferentiated astrocytes have used serum containing media (Meyer et al., 2014; Varcianna et al., 2019) and so the feasibility of this approach in serum free conditions remains to be comprehensively demonstrated.

### 1.2 Human *in vitro* microglia models

Several human *in vitro* microglial models have been developed in recent years (Table 3) (Hasselmann and Blurton-Jones, 2020; Sabogal-Guáqueta et al., 2020). Cells obtained from these protocols display many microglial specific genes and carry out microglial functions such as phagocytosis and cytokine release in response to proinflammatory stimulation. However, several of these protocols produce macrophages which have not been validated as independent of the transcription factor MYB, a key determinant of the correct ontogeny to yolk sac-derived fetal macrophages (Schulz et al., 2012), and vary in the enrichment of microglia obtained (Buchrieser et al., 2017; Haenseler and Rajendran, 2019). Some protocols instead have distinguished microglia from macrophages by calcium responses to adenosine diphosphate (Douvaras et al., 2017), with respiratory bursts or displaying contact inhibition *in vitro* (Nimmerjahn et al., 2005; Pocock and Piers, 2018). Microglial specific markers P2RY12 or TMEM119 have also been used but this may not be reliable due to changes in different reactive states and the acquisition of certain macrophages expressing microglial specific markers upon their entry into the CNS during chronic inflammation (Grassivaro et al., 2020).

**Table 3:** Summary of different human *in vitro* microglia protocols.

Citation Reference	Summary
Etemad et al. (2012)	Primary monocytes cultured with M-CSF, GM-CSF, NGF and CCL2 acquire a ramified morphology and lower levels of CD45, CD14, HLA-DR, CD11b and CD11c.
Ohgidani et al. (2014)	Primary monocytes converted to microglia-like cells with incubation of GM-CSF and IL-34 to a more ramified morphology.
Muffat et al. (2016)	Microglia-like cells expressing TMEM119, from hESCs and hiPSCs. EBs cultured on murine embryonic fibroblast feeders before differentiation with M-CSF and IL-34. Can co-culture in 3D with neurons and astrocytes and they respond to LPS with cytokine secretion.
Abud et al. (2017)	Initially hiPSCs cultured in 5% oxygen conditions during haematopoiesis, then M-CSF, IL-34 and TGF-β followed by maturation with CD200 and CX3CL1 and then FACS for CD43. Microglia-like cells exhibit cytokine secretion, cell migration, responses of calcium and phagocytosis.
Douvaras et al. (2017)	Microglia-like cells isolated by FACS for CD14 and CX3CR1. Microglia-like cells express IBA1, CD11c, TMEM119, P2RY12, CD11b and CX3CR1. Release cytokines after LPS/IFNγ treatment, phagocytose and are calcium responsive after ADP treatment.
Haenseler et al. (2017a,b)	MYB-independent hiPSC derived microglia are motile and phagocytotic when co-cultured with iPSC derived cortical neurons and secrete cytokines upon treatment with LPS.
Pandya et al. (2017)	hiPSCs differentiated to microglia-like cells with GM-CSF, M-CSF, and IL-3 on astrocyte monolayers before being MACS sorted for CD11b/CD39. Express HLA-DR, CD45, TREM-2 and CX3CR1 but are negative for CD86, CD206, CD200R and CD80. Phagocytose and secrete TNF-α after LPS treatment.
McQuade et al. (2018)	Updated simplified Abud et al., protocol without the need for 5% oxygen or cell sorting.

hiPSCs can be directed to mesodermal cell lineage through application of BMPs, VEGF and/or SCF. This is then followed by application of ligands such as M-CSF, IL-3, IL-34 and additional factors such as CD200 and CX3CL1 to promote microglial differentiation (Muffat et al., 2016; Abud et al., 2017; Douvaras et al., 2017; Haenseler et al., 2017a; Pandya et al., 2017; McQuade et al., 2018). Some protocols use cell sorting to obtain pure cultures of microglia, although this is not required for high purity (Abud et al., 2017; Douvaras et al., 2017; Pandya et al., 2017). Other protocols attempt to consolidate microglial identity through co-culture with neurons or astrocytes (Muffat et al., 2016; Haenseler et al., 2017a; Pandya et al., 2017). No direct comparison has been made between different protocols, but it is clear that extended incubation with microglial associated ligands,co-culture with CNS cell types, or application of neural precursor conditioned media (Banerjee et al., 2020) aids the consolidation of a microglial phenotype. Human microglia developed *in vitro* have been shown to successfully engraft into mice brains, retain their human microglial identity and perform key functions including synaptic pruning (Mancuso et al., 2019; Svoboda et al., 2019; Xu et al., 2020).

In addition to hiPSC based microglial protocols, human microglia have also been modelled using primary human monocytes (Etemad et al., 2012) and immortalised cell lines (Tsuchiya et al., 1980; Janabi et al., 1995). These cells can be obtained faster than hiPSC based protocols but are limited in terms of their supply or experience karyotype abnormalities, respectively. Furthermore, both of these methods lack the important distinct ontological background of microglia. Some studies have attempted to address this limitation by treating primary cells with factors used to promote microglial differentiation. Treatment of primary human peripheral blood cells with Granulocyte-macrophage colony-stimulating factor (GM-CSF), M-CSF, Chemokine (C-C motif) ligand 2 (CCL2), Nerve growth factor (NGF-β) and IL-34 induced a ramified morphology with increased expression of microglial specific genes such as *TGFBR1*, *PROS1*, *P2RX7* and *C1QB* (Etemad et al., 2012; Ryan et al., 2017). Furthermore, treatment with only IL-34 and GM-CSF has been shown to be sufficient to recapitulate a more microglial identity with increased CX3CR1 and decreased CD45, CD14 and CCR2 (Ohgidani et al., 2014) (Table 4).

**Table 4:** Advantages and disadvantages of human *in vitro* microglia models.

Model	Strengths	Limitations
hiPSC	High purity	Expensive
	Ability to self-renew	Time consuming
		Developmental model
Primary macrophages	Can obtain from adult donors	Limited supply
		Different ontogeny
Immortalised cell line	Fast	Karyotype abnormalities
	Ability to self-renew	Abnormal proliferative state
		Currently reliant on serum affecting reactivity

## 2 Reactive transformation of hiPSC derived astrocytes and microglia

Astrocytes and microglia are able to undergo dramatic changes in gene expression in response to a wide array of stimuli in a process termed reactive gliosis. These states have been classified as a continuum between either M1/A1 or M2/A2 depending on their neurotoxic or neuroprotective attributes, respectively. However, this classification may be an oversimplification as a range of reactive states is likely to exist expressing both protective and detrimental markers in a non-mutually exclusive manner (Ransohoff, 2016; Liddelow and Barres, 2017). Reactive states may be transient and are likely to be temporally regulated depending on the chronicity of the stimulus and the age of the cell. Human *in vitro* models have further demonstrated the cellular autonomy of astrocyte and microglial reactive processes, as highly purified populations of these cell types can be obtained that are naive to interactions with other cell types.

hiPSC derived astrocytes respond differentially to different cytokines in terms of their transcriptional profiles and secretion of factors (Perriot et al., 2018; Thelin et al., 2020). As previously mentioned above, it is important to note that the presence of serum has been associated with the transformation to an irreversible reactive state and thus studies investigating reactivity should be performed under serum-free conditions (Foo et al., 2011; Perriot et al., 2018). The adoption of an A1 reactive state through stimulation with TNF-α, IL-1α and C1q has been recently recapitulated in an important comprehensive study using hiPSC derived astrocytes (Barbar et al., 2020). In this study, it was demonstrated that upon adoption of an A1 reactive state, astrocytes release proinflammatory factors whilst simultaneously losing homeostatic functions such as phagocytosis and glutamate uptake. It is currently unclear whether this increase in secretion of factors and the loss of homeostatic functions are mechanistically linked or merely correlated with one another.

hiPSC derived microglia have also been demonstrated to undergo reactive changes in response to inflammatory stimuli. Stimulation of microglia with lipopolysaccharide (LPS)/Interferon gamma (IFN-γ) resulted in morphological changes and cytokine release (Haenseler et al., 2017a; Garcia-Reitboeck et al., 2018). However, the impact of reactive stimulation on other functions of microglia is lacking. Furthermore, while both hiPSC derived astrocytes and microglia have been found to become reactive when stimulated with pro-inflammatory factors, the effects of anti-inflammatory stimuli on morphology, gene expression and function remain unexplored. It is important to note that both astrocyte and microglial hiPSC protocols may affect subsequent reactivity. Other than the effects of serum on reactive state, other factors including LIF, TGF-β and M-CSF used in published protocols have been associated with affecting reactive profiles of astrocytes and microglia (Gowing et al., 2009; Sofroniew and Vinters, 2010; Cekanaviciute et al., 2014).

## 3 Using human *in vitro* models to study astrocytes and microglia in neurodegeneration

While neurodegenerative diseases are characterized by the loss of specific neuronal populations, dysfunction of both astrocytes and microglia have been heavily implicated in contributing to neuronal death. Several genes which are highly expressed in astrocytes and/or microglia are linked to neurodegenerative diseases including *APOE4*, *TREM2*, *CR1* and *C9ORF72*. Furthermore, prion-like spread of pathological proteins between neurons and glia has been reported including Amyloid-β (Aβ) in Alzheimer’s disease (AD) (Veeraraghavalu et al., 2014), α-synuclein in Parkinson’s disease (PD) (Lee et al., 2010; Loria et al., 2017), Huntingtin in Huntington’s disease (HD) (Pearce et al., 2015; Donnelly et al., 2020) and TAR DNA-binding protein 43 (TDP-43) in Amyotrophic Lateral Sclerosis (ALS) (Smethurst et al., 2020). Human *in vitro* models of astrocytes and microglia are able to capture some phenotypes of these neurodegenerative diseases and demonstrate that a particular phenotype is cell autonomous. Furthermore, findings from human *in vitro* models of neurodegenerative diseases have begun to shed light on the primacy of pathological events. With many genetic traits of neurodegenerative diseases occurring in glial cells, it is unsurprising that much focus has been on modelling astrocytes and microglia derived from patients with identified disease-linked mutations. iPSC-derived human *in vitro* models have provided valuable insight into the pathogenic mechanisms that underlie neurodegenerative disease, but remain limited in the study of sporadic cases.

### 3.1 Alzheimer’s disease

**Fig. 1:**
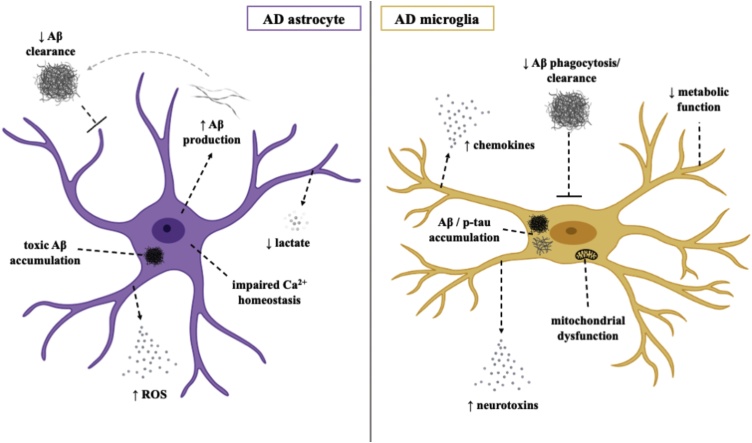
Pathomechanisms of AD in human *in vitro* astrocytes and microglia.

AD is the most common cause of dementia worldwide and is characterised by progressive decline in cognitive functions, particularly episodic memory. AD neuropathology includes the presence of extracellular Aβ plaques and intraneuronal neurofibrillary tangles made up of hyperphosphorylated Tau, both contributing to synaptic dysfunction and subsequent neurotoxicity, found throughout the cerebral cortex and sub-cortical regions in the post-mortem brain (Braak and Braak, 1991). Many of the GWAS-identified loci that confer genetic risk for late-onset AD are highly expressed in astrocytes and microglia, with the first identified and most studied being the *APOE4* allele of the polymorphic *APOE4* gene (Corder et al., 1993). Physiological Apolipoprotein E (APOE) serves as a lipid carrier and helps to support synaptic integrity and promote neuronal survival (Liu et al., 2013). With its primary source of expression being astrocytes, and to a lesser extent microglia and neurons, the *APOE4* variant has been shown to disrupt physiological glial cell function, highlighting a potential mechanism by which astrocytes and microglia are involved in AD pathogenesis. Phenotypes associated with AD that have been identified in human *in vitro* models of astrocytes and microglia are summarised in Fig. 1.

#### 3.1.1 Astrocytes

Astrocytes are crucial in the clearance of metabolites and toxins, including Aβ. In response to increasing levels of Aβ in the AD brain, astrocytes become reactive and undergo functional changes that impact clearance mechanisms, thus compromising synaptic and neuronal viability (Abramov et al., 2003; Rowland et al., 2018). In addition to the contribution of Aβ accumulation through defective clearance, reactive astrocytes have also been implicated in the production of Aβ possibly through an increase in β-Secretase 1 activity (Oksanen et al., 2017) a rate-limiting enzyme that catalyses the initial cleavage of Amyloid precursor protein (APP). While reactive astrogliosis represents an additional hallmark of AD, and one that precedes neuronal death, the direct contribution to disease progression remains unclear.

Such astrocyte pathology in AD has been recapitulated through various human *in vitro* iPSC models. iPSC-derived astrocytes from both familial and sporadic AD patients have been shown to exhibit disease phenotypes, including global morphological abnormalities and aberrant secretion of several factors (Jones et al., 2017). In an important study, hiPSC derived astrocytes generated from patients with *PSEN1* exon 9 deletions exhibited increased Aβ production, impaired cytokine release, overproduction of reactive oxide species and disrupted calcium metabolism (Oksanen et al., 2017). Similar stress-induced changes were also reported in an hiPSC derived astrocyte model from AD patients with *APP* mutations, suggested to be attributed specifically to intracellular accumulation of Aβ oligomers (Kondo et al., 2013). Morphological abnormalities, namely generalized atrophy, and altered expression of astrocytic markers have also been described in hiPSC derived astrocytes from *PSEN1*^M146L^ and *APOE4*^+/+^ patients (Jones et al., 2017; Lin et al., 2018). APOE4-hiPSC derived astrocytes were also shown to be less efficient in Aβ uptake and clearance than APOE3-astrocytes, an effect that correlated with changes in lipid metabolism (Lin et al., 2018). Importantly, conversion of APOE4 to APOE3 in a sporadic AD line enhanced the ability of both hiPSC derived astrocytes and microglia to perform Aβ uptake. Another recent study using hiPSC derived astrocytes to explore the role of APOE4 in AD pathogenesis demonstrated marked reduction in supportive neurotrophic and synaptogenic functions in *APOE4*^+/+^ astrocytes compared to those with an *APOE3*^+/+^ profile (Zhao et al., 2017).

#### 3.1.2 Microglia

Aβ accumulation seems to instigate prolonged recruitment and clustering of microglia at the site of senile plaques in the AD brain. While microglia express receptors implicated in Aβ clearance and phagocytosis (Simard et al., 2006), such as CD36, and RAGE (Yan et al., 1996; El Khoury et al., 1998) why Aβ continues to accumulate and AD pathology progresses despite recruitment of supposedly protective cells remains unclear. However, evidence points to a combination of dysfunctional Aβ-clearing mechanisms (Hickman et al., 2008) and Aβ-induced activation of proinflammatory processes through upregulated secretion of cytokines and neurotoxins (Guillot-Sestier and Town, 2013). Microglial expression of known AD risk factors such as *TREM2* and *CD33* further emphasises a likely role microglia play in AD pathogenesis (Abud et al., 2017). A recent hiPSC derived microglial model from healthy patients demonstrated some physiological properties of microglia, including phagocytosis of Aβ, tau oligomers and human synaptosomes (Abud et al., 2017), providing a suitable platform to uncover pathogenic pathways.

A recent study comparing hiPSC derived microglia from AD patients and cognitively normal age-matched controls reported stronger phagocytic function in AD-microglia with or without a LPS induced inflammatory stimulus (Xu et al., 2019). High concentrations of LPS were significantly less toxic to AD-microglia and led to increased cytokine secretion, a phenotype that mimics pathological changes in AD microglia (Xu et al., 2019). Increased reactive oxide species has also been observed in hiPSC microglia from sporadic AD patients, as well as increased phagocytosis in response to H_2_O_2_ treatment (Zhang et al., 2020). When compared to those from *PSEN1*^ΔE9^ and *APP* mutation backgrounds, microglia from *APOE4*^+/+^ patients exhibit significantly impaired phagocytosis, migration and metabolic function as well as increased cytokine production (Konttinen et al., 2019). To ascertain the effect of APOE4 on AD pathology in a 3D system, hiPSC derived microglia from *APOE3* and *APOE4* backgrounds were co-cultured with *APP* duplication organoids (Lin et al., 2018). Identification of longer microglial processes, heightened levels of phosphorylated Tau and increased Aβ deposition in *APOE4*-organoids suggested that the APOE4^+/+^ genotype confers microglial dysfunction through reduced clearance of Aβ (Lin et al., 2018).

*TREM2* represents another genetic risk factor associated with a greater risk of developing AD. *TREM2* knockout hiPSC microglia have been shown to elicit increased apoptotic cell death and impaired phagocytic ability (Claes et al., 2019; Hall-Roberts et al., 2020; McQuade et al., 2020). In addition, *TREM2* knockout microglia transplanted into a mouse model AD failed to effectively migrate and cluster around Aβ plaques. Significant metabolic deficits including mitochondrial dysfunction and an inability to carry out a glycolytic immunometabolic switch have been observed in *TREM2*^R47H^ hiPSC derived microglia, due to dysregulation in PPARγ/p38MAPK signaling pathways (Piers et al., 2020). Activation of these pathways ameliorated these deficits and rescued microglial protective functions such as Aβ phagocytosis (Piers et al., 2020). However, other studies have found no dysfunction in phagocytic ability in *TREM2*^R47H^ human *in vitro* models of microglia (Claes et al., 2019; Hall-Roberts et al., 2020).

A 3D human tri-culture model of AD enabling the investigation of the important interactions between neurons, astrocytes and microglia has been recently developed (Park et al., 2018). This was achieved by overexpression of mutant APP in neural progenitor cells to produce neurons and astrocytes before immortalised adult microglia were added. Increased Aβ and phosphorylated Tau were observed in the 3D system compared to a 2D system of mutant APP neurons and astrocytes. Microglia cultured with APP mutant neurons and astrocytes became more motile in a CCL2 dependent process and released a number of pro-inflammatory cytokines and chemokines. Furthermore, microglia in this setting induced the death of neurons and astrocytes in a process partly dependent on TLR4 and IFN-γ. The development of this model has provided a unique opportunity to better understand cellular interplay in the CNS as well as the influence of cell specific effects of AD-associated mutations and the potential interactions between different mutations occurring in astrocytes, microglia and neurons.

### 3.2 Parkinson’s disease

PD is the second most prevalent neurodegenerative disease, affecting both motor and non-motor functions and is characterized by substantial loss of dopaminergic neurons in the substantia nigra pars compacta. While the distinguishing neuropathological hallmark of PD is the presence of Lewy bodies, made up of misfolded and abnormally aggregated α-synuclein, evidence of inflammatory activity in the CNS is another major pathological feature of PD including activation of both astrocytes and microglia (Imamura et al., 2003; Miklossy et al., 2006; Doorn et al., 2014).

**Fig. 2:**
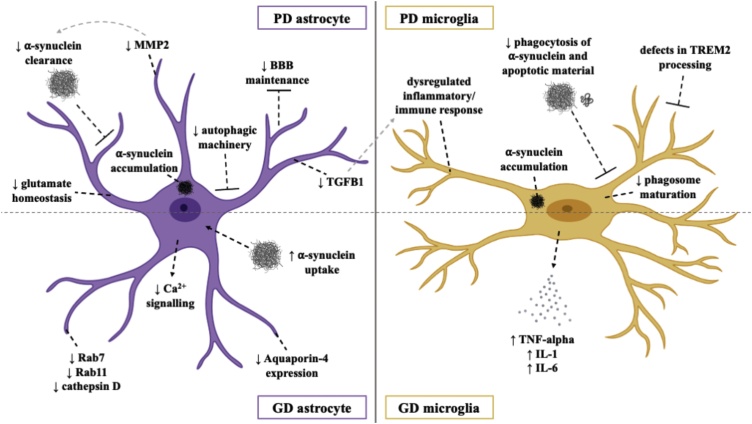
Pathomechanisms of PD in human *in vitro* astrocytes and microglia.

Mutations in Leucine-rich repeat kinase 2 (LRRK2) are known to cause autosomal dominant and sporadic PD, with the majority of mutations occurring within enzymatic domains. Such mutations have been found to upregulate inherent LRRK2 kinase activity and reduce GTPase activity (Ferreira and Massano, 2017). With LRRK2 variants also being associated with autoimmune and infectious diseases, it is unsurprising that LRRK2 is expressed in numerous immune cells such as microglia (Moehle et al., 2012; Lee et al., 2020). Studies using hiPSCs to investigate PD pathogenesis have focused predominantly on mechanisms underlying the degeneration and death of dopaminergic neurons. Studies using hiPSCs generated from PD patients with LRRK2 mutations have described the appearance of disease-specific phenotypes in hiPSC derived neurons, including impaired axonal outgrowth, increased susceptibility to oxidative stress (Nguyen et al., 2011), pathogenic activation of the unfolded protein response (Heman-Ackah et al., 2016) and deficient autophagic vacuole clearance (Sánchez-Danés et al., 2012). Phenotypes identified in human *in vitro* astrocyte and microglial models of PD are detailed below (Fig. 2).

#### 3.2.1 Astrocytes

Post-mortem discovery of astrocytic accumulation of α-synuclein in the PD brain (Wakabayashi et al., 2000; Braak et al., 2007) prompted a shift in focus to the role of astrocyte dysfunction in PD pathogenesis. A myriad of recent studies have supported the emerging phenomena of pathogenic α-synuclein transfer and accumulation *via* aberrant neuron-astrocyte interactions, disruption to normal astrocytic function and subsequent loss of neuronal viability (Gu et al., 2010; Lee et al., 2010; Booth et al., 2017; Cavaliere et al., 2017). Such functional disruptions include impaired autophagy (di Domenico et al., 2019), glutamate homeostasis and blood-brain-barrier maintenance (Gu et al., 2010).

A recent notable study employed a co-culture system of hiPSC derived astrocytes from patients with PD-causing LRRK2 mutations and healthy hiPSC derived dopaminergic neurons to explore the pathogenic impact on neuron-astrocyte crosstalk. LRRK2 mutant astrocytes elicited a significant reduction in the survival rate of dopaminergic neurons, coupled with defective autophagic machinery and abnormal accumulation of endogenous α-synuclein (di Domenico et al., 2019). In the same study, treatment with an activator of chaperone-mediated autophagy ameliorated dysfunction of this process and prevented the manifestation of disease phenotypes in PD astrocytes. Another study, also using hiPSCs derived from PD LRRK2-positive patients, reported astrocyte-specific downregulation of genes associated with inhibition of microglial inflammatory responses and α-synuclein aggregate degradation (Booth et al., 2019), importantly implicating the loss of astrocyte neuroprotective capacity as a key player in the development of PD pathology.

Gaucher disease (GD) is a lysosomal storage disorder elicited by mutations in the gene that encodes the Glucocerebrosidase beta 1 (GBA1) enzyme, also representing a critical risk factor for PD and the related dementia with Lewy bodies (Sidransky et al., 2009; Nalls et al., 2013). Using hiPSC derived astrocytes from patients with GD type 1 (non-neuronopathic with and without PD) and type 2 (neuronopathic), a recent study reported astrogliosis in GD2-astrocytes as measured by increased GFAP and marked reduction in Aquaporin-4 expression (Aflaki et al., 2020) a channel implicated in blood-brain-barrier function, water transport and astrocyte migration (Nagelhus and Ottersen, 2013). GD type 1 and 2 hiPSC derived astrocytes also displayed dysfunctional calcium signalling and when incubated with α-synuclein monomers or fibrils showed increased uptake and reduced expression of endosomal markers RAB7 and RAB11 and lysosomal marker Cathepsin D, suggesting compromised α-synuclein clearance (Aflaki et al., 2020). PD and GD-associated astrocyte and microglial phenotypes identified through human *in vitro* models can be seen in Fig. 2.

#### 3.2.2 Microglia

Microglial phenotypes have also been reported in hiPSC models of PD. An hiPSC derived microglia-like macrophage model from an early-onset PD patient with a *SNCA* triplication demonstrated a significant increase in both intracellular and extracellular α-synuclein when compared to controls (Haenseler et al., 2017b), mirroring effects seen in an hiPSC derived astrocyte model of PD. This study also reported altered expression of CXCL1, IL-18 and IL-22 and reduced phagocytic competence in PD microglial-like cells, which was replicated in control macrophages upon addition of monomeric α-synuclein to the culture medium (Haenseler et al., 2017b). Together, these findings suggest that a pathogenic build-up of α-synuclein in microglia-like cells results in a loss of function mechanism where the inherent clearance properties of microglia are jeopardised.

Recently, an elegant study uncovered a link between inflammation and neurodegeneration in PD using hiPSC-derived dopaminergic neurons and microglia carrying the LRRK2^G2019S^ mutation (Panagiotakopoulou et al., 2020). It was demonstrated that LRRK2^G2019S^ microglia display increased motility, phagocytic capacity and abnormal metabolic activity and immune responses to IFN-γ or LPS stimulation. Furthermore, LRRK2^G2019S^ but not control microglial conditioned media treated with LPS reduced neurite length of hiPSC dopaminergic neurons, directly implicating altered microglial immune responses in neuronal dysfunction in PD.

Another study exploring LRRK2 function in hiPSC derived macrophages found genetic knockout or pharmacological inhibition of LRRK2 impeded phagosome maturation, contributing to mycobacterial replication and dampening innate immune responses (Härtlova et al., 2018). Using an hiPSC derived *LRRK2* knockout microglial model and an isogenic control line, a more recent study highlighted a specific role of LRRK2 in the recruitment of RAB8a and RAB10 to phagosomes, identifying its function at the junction between phagosome maturation and recycling pathways (Lee et al., 2020). PD relevant microglial phenotypes have also been described in hiPSC microglia-like macrophages derived from GD patients with *GBA1* mutations including elevated expression of TNF-α, IL-6 and IL-1, further exacerbated by LPS-stimulation (Panicker et al., 2012, 2014).

### 3.3 Huntington’s disease

**Fig. 3:**
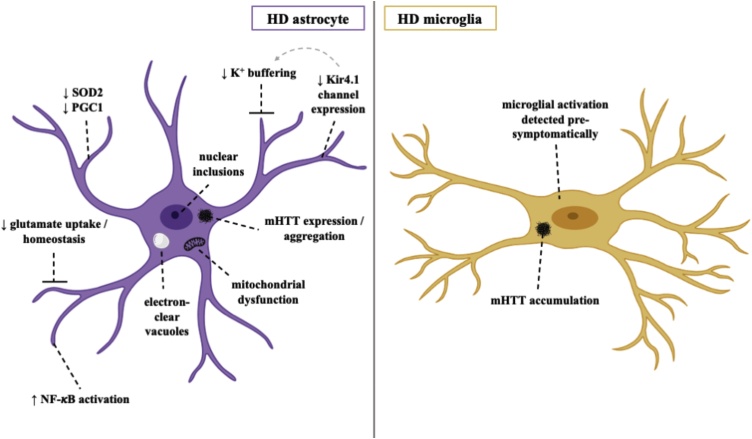
Pathomechanisms of HD in human *in vitro* astrocytes and microglia.

HD is a rare and rapidly progressive hereditary neurodegenerative disorder caused by CAG repeat expansions in the Huntingtin (*HTT*) gene, accumulation of its mutant protein (mHTT) (Bates et al., 2015) and consequent detriment to glial health and neuronal survival. Such neuropathology is most prevalent in the neostriatum of the HD brain, with marked loss of neostriatal medium spiny neurons (Benraiss et al., 2016). Several causative mechanisms have been proposed for this HD-specific degeneration profile, including non-cell-autonomous contribution from astrocytes (Juopperi et al., 2012) (Fig. 3).

#### 3.3.1 Astrocytes

Mounting evidence implicating both reactive astrocytes and microglia in HD pathogenesis has led to the publication of many studies exploring their dysfunction, primarily in rodent models. Engraftment of mHTT-expressing human glial progenitor cells has been shown to inflict a disease phenotype on healthy mice, with healthy glia alleviating such effects in transgenic HD mice (Benraiss et al., 2016). Expression of mHTT in mouse astrocytes specifically has been shown to induce functional atrophic changes that may drive excitotoxicity, such as impaired glutamate homeostasis (Liévens et al., 2001; Bradford et al., 2009; Jiang et al., 2016), mitochondrial dysfunction (Schon and Przedborski, 2011) and diminished capacity to buffer extracellular potassium through downregulation of a striatum-specific Kir4.1 potassium channel (Tong et al., 2014). As with other neurodegenerative disorders, dysregulation in astrocytic neuroinflammatory signaling pathways is also implicated in HD pathogenesis such as chronic activation of NF-κB (Hsiao et al., 2013). While these findings are undeniably useful, few studies have investigated such effects in human astrocyte models of HD.

hiPSC derived HD astrocytes provide less functional support for neuronal maturation when co-cultured with HD and control neurons and are unable to protect neurons as effectively from glutamate-induced cytotoxicity (Garcia et al., 2019). Using hiPSC derived astrocytes from a father and daughter with adult onset and juvenile HD respectively revealed a unique vacuolation phenotype (Juopperi et al., 2012). This disease-specific astrocytic phenotype, marked by the presence of cytoplasmic electron-clear vacuoles, had previously only been observed in peripheral blood lymphocytes from HD patients, thus suggesting a potential role of vacuolation in HD pathogenesis (Juopperi et al., 2012). In a more recent study, an astrocyte differentiation protocol from hiPSCs of human oligomeric mHTT-injected HD monkeys was developed (Cho et al., 2019). Consistent with previous findings, astrocytic expression of mHTT induced a plethora of downstream pathogenic effects, including cytosolic mHTT aggregation and nuclear inclusion formation, impaired glutamate uptake capacity and down-regulated expression of SOD2 and PGC1 (Cho et al., 2019). Importantly, successful amelioration and reversal of such HD astrocyte-specific phenotypes was observed upon expression of anti-HTT small-hairpin RNA molecules. While there is undeniable demand for further study, these hiPSC-derived HD models collectively highlight the critical influence of mHTT on human astrocytes, thus challenging our view on the exclusivity of neuron-focused disease mechanisms and bringing alternative drug targets to light.

#### 3.3.2 Microglia

The identification of reactive microglia in the neostriatum, cortex and globus pallidus of human HD brain tissue sparked interest in the role of microglia and neuroinflammation in HD pathogenesis, with the number of reactive microglia directly correlating with degree of neuronal cell death (Sapp et al., 2001). A correlation between microglial activation and disease severity has been described in both HD mice and patients (Pavese et al., 2006; Simmons et al., 2007), which when considered together with microglial suppression correlating with the prolonged lifespan of HD mice (Zwilling et al., 2011) reinforces microglial involvement in HD. The aberrant accumulation of mHTT in microglia has also been considered an integral component of progressive neurodegeneration in HD, with their activation detected pre-symptomatically in HD carriers as well as in the post-mortem HD brain (Yang et al., 2017). To the authors’ knowledge, however, a viable hiPSC derived microglia model for HD has yet to be reported.

### 3.4 Amyotrophic lateral sclerosis and frontotemporal dementia

**Fig. 4:**
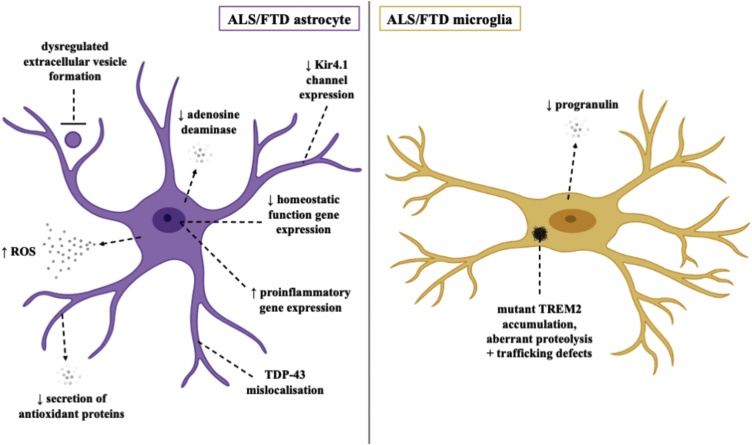
Pathomechanisms of ALS/FTD in human *in vitro* astrocytes and microglia.

ALS is a rapidly progressing disease characterized by the degeneration of upper and lower motor neurons (Hardiman et al., 2017). Overlap of clinical, cellular and genetic aspects of disease with Frontotemporal dementia (FTD), a disease characterised by progressive cognitive decline and behavioural abnormalities, has reframed ALS and FTD as part of a disease spectrum. Several genes encoding proteins with diverse cellular functions have been implicated as causative for ALS including *C9ORF72*, *SOD1*, *TARDBP, FUS* and *VCP*. Both astrocytes and microglia have been implicated as important modulators of the pathological process of motor neuron death in ALS in animal models of the disease (Boillée et al., 2006; Yamanaka et al., 2008). Human *in vitro* models of astrocytes have recapitulated some of the phenotypes observed in human post-mortem tissue and animal models of ALS, while investigation of ALS human *in vitro* microglia models is currently lacking (Fig. 4).

#### 3.4.1 Astrocytes

The neuroprotective capacity of hiPSC derived astrocytes to motor neurons has been recently demonstrated using sporadic ALS (sALS) spinal cord extracts to seed aggregation of TDP-43 into hiPSC derived motor neurons (Smethurst et al., 2020). In this study, astrocyte conditioned media reduced mislocalization and aggregation of cytoplasmic TDP-43 and improved motor neuron survival.

hiPSC derived astrocytes harbouring ALS causing mutations have been shown to recapitulate some ALS phenotypes and be detrimental to co-cultured neurons. TDP-43 mutant astrocytes have been shown to display TDP-43 mislocalisation (Serio et al., 2013). While these mutant astrocytes did not affect the survival of co-cultured neurons, an important observation was that mutant astrocytes undergo cell death themselves. While TDP-43 mutant astrocytes did not cause the death of co-cultured neurons, ALS causing Valosin-containing protein (VCP) mutant astrocytes exhibited disrupted support of motor neuron survival in co-culture in addition to a cell autonomous survival phenotype (Hall et al., 2017). Furthermore, in an important study utilising astrocytes derived from transdifferentiated human fibroblasts from both sporadic and familial ALS cases including mutant Superoxide dismutase 1 (SOD1) and Chromosome 9 open reading frame 72 (C9ORF72), also were toxic to co-cultured motor neurons (Meyer et al., 2014). sALS astrocytes transplanted into the spinal cords of immunodeficient mice induced motor neuron death and motor deficits (Qian et al., 2017).

Astrocytes are thought to contribute to ALS pathology through both a transformation to a toxic reactive state and through the loss of homeostatic functions. In SOD1 mutant astrocytes derived from hiPSCs, many proinflammatory genes were increased while several genes associated with homeostatic functions were decreased (Tyzack et al., 2017). SOD1 mutant astrocytes also express decreased levels of Kir4.1, which is critical in regulating fast firing motor neuron function and may partly account for the specific vulnerability of fast firing motor neurons in ALS (Kelley et al., 2018). Downregulation of the adenosine deaminase was found in astrocytes transdifferentiated from C9ORF72 ALS human fibroblasts, correlating with increased toxicity of co-cultured motor neurons likely due to an increase in the substrate, adenosine, and decrease in product, inosine (Allen et al., 2019). These astrocytes have also been reported to display dysregulated extracellular vesicle formation (Varcianna et al., 2019). In this study, C9ORF72 astrocyte extracellular vesicles recapitulated toxicity of conditioned media to motor neurons, which may be due to the dysregulation of miRNAs such as miR-494-3p.

There has been some controversy over the toxicity of C9ORF72 ALS hiPSC derived astrocytes, with one group reporting loss of co-cultured hiPSC derived motor neuron electrophysiological function but no effect on viability (Zhao et al., 2020). However, other groups found that hiPSC derived C9ORF72 astrocyte conditioned media was toxic to both hiPSC derived motor neurons and mouse cortical neurons, possibly due to the acquisition of a senescent phenotype, decreased secretion of antioxidant proteins and increased reactive oxide species production (Madill et al., 2017; Birger et al., 2019). Further studies using the same lines with isogenic controls and standardized differentiation paradigms will help to reconcile these seemingly divergent results.

#### 3.4.2 Microglia

Very few studies have investigated phenotypes of ALS/FTD in human *in vitro* models of microglia. hiPSC derived microglia have been developed from patients with FTD causing progranulin mutations (Almeida et al., 2012), however, other than reduced progranulin levels no phenotypes have been reported. hiPSC derived microglia have been generated from lines of the FTD-like syndrome, Nasu Hakola disease, which is due to missense mutations in TREM2 (Brownjohn et al., 2018). However, although deficits in TREM2 were observed in microglia, no functional defects in phagocytosis or secretion of inflammatory cytokines IL-6, TNF-α and IL-1β in response to LPS treatment were observed.

## 4 Conclusions

The advent of human *in vitro* models of astrocytes and microglia has opened exciting new possibilities for disease modelling and drug screening. Both astrocyte and microglia human *in vitro* models partially recapitulate the transcriptomes of their *in vivo* counterparts and are able to capture authentic disease phenotypes. Further investigation of monocultures to resolve cellular autonomy in disease, as well as co-culture paradigms between astrocytes and microglia and between neurons and glia will provide a deeper insight into the cellular interplay of different diseases and may lead to the development of cell type specific therapies.

## Funding information

This work was supported by the 10.13039/100010438Francis Crick Institute which receives its core funding from 10.13039/501100000289Cancer Research UK (FC010110), the UK Medical Research Council (FC010110), and the 10.13039/100010269Wellcome Trust (FC010110). R.P. holds an MRC Senior Clinical Fellowship [MR/S006591/1].
