# Supplementary material for: Astrocytes and microglia in neurodegenerative diseases: Lessons from human in vitro models
Source: Prog Neurobiol. 2021 May;200:101973. doi: 10.1016/j.pneurobio.2020.101973 (PMC8052192; doi:10.1016/j.pneurobio.2020.101973)
Supplement: Supplementary file 1 [file mmc1.pdf]

Peer Review Overview

Manuscript Title: “Astrocytes and microglia in neurodegenerative diseases: lessons from human in vitro models”

Received	03-Sep-2020
1st Decision	26-Oct-2020
Revision Submitted	06-Nov-2020
Accepted	05-Dez-2020

Decision Letter

Dear Professor Patani,

Thank you for submitting your manuscript to Progress in Neurobiology. We have received comments from reviewers on your manuscript. Your paper should become acceptable for publication pending suitable minor revision and modification of the article in light of the appended reviewer comments.

When resubmitting your manuscript, please carefully consider all issues mentioned in the reviewers' comments, outline every change made point by point, and provide suitable rebuttals for any comments not addressed.

To submit your revised manuscript go to https://www.editorialmanager.com/proneu/ and log in as an Author where you will see a menu item called 'Submission Needing Revision'.

Please resubmit your manuscript by Dec 25, 2020.

We look forward to receiving your revised manuscript.

Kind regards,

Aimee Kao
Associate Editor
Progress in Neurobiology

Sabine Kastner
Editor-in-Chief
Progress in Neurobiology

Comments from the Editors and Reviewers:

Reviewer #1: This review article by Franklin et al. nicely summarizes recent works using human in vitro models of astrocytes and microglia, and how they have been used to understand new aspects of glial involvement in neurodegenerative diseases. Overall, the manuscript is well written and provides a nice background on in vitro culture models, as well as the contribution of genetic variants to different neurodegenerative diseases. The tables and figures appropriately complement the text. Some minor comments are offered:

1) In Tables 2 and 4, karyotype abnormalities are listed as the only limitations for immortalized cell lines. Additional limitations of immortalized astrocytes and microglia could be listed here.
2) In section 1, it is mentioned that inclusion of serum in astrocyte culture can have a profound influence on baseline reactivity. The matrix on which the cells are cultured can also contribute to the baseline properties of the cells, and it would be beneficial for the authors to provide some discussion on this point.
3) The sentence "Furthermore, hiPSC derived astrocytes are only partially mature and are currently unable to capture age-related phenotypes" could be elaborated.
4) "Fluorescence activated cell sorting (FACS) can be used to purify cultures (Barbar et al., 2020),

however, this is not necessary to obtain highly pure cultures" This statement could be further explained. What is considered sufficient purity for studies?
5) "However, several of these protocols produce macrophages which have not been validated as independent of the transcription factor MYB and vary in the yield of microglia obtained" Please explain the importance of MYB here.
6) "Astrocytes and microglia are able to undergo dramatic changes in gene expression in response to a wide array of stimuli in a process termed astrocyte reactivity". Consider subbing "astrocyte reactivity" with "reactive gliosis" which is inclusive of both astrocytes and microglia.
7) The disease section mostly focuses on genetic variants that either cause or increase risk for the neurodegenerative diseases discussed. Additional commentary could be provided on how astrocytes/microglia can be/have been used to study non-genetic contributions.

Reviewer #2: The review of Franklin et al. gives a nice overview of the potential involvement of astrocytes and microglia in different neurodegenerative disorders. In addition to an interesting overview of the differences between astrocytes and microglia and the different methods to obtain the different in vitro models, the review mainly concentrates on those phenotypes that were obtained using human in vitro models in different neurodegenerative disorders. The review is well structured, very well written and summarises a lot of information. It could be interesting for a very broad audience.

Major remarks
- The review is rather descriptive. It doesn't offer clear insights into what the human in vitro models exactly contributed to the different research fields. Apart from an overview of the obtained results, the review could have an important added value if a clear distinction could be made between occasional observations and important new insights and/or concepts which could potentially lead to completely new strategies to interfere with the different neurodegenerative diseases. It might be too early to come to such a conclusion (see also next remark).
- While the review mainly focuses on the phenotypes observed in the different in vitro models, information on the overall limitations of these in vitro models is lacking. At present, limitations are limited to the different model systems (Tables 2 and 4).
- The quality and added value of the figures is limited. Text is too small and the added value is not so clear as it is mainly listing the mechanistic insights described in the text with lines pointing to the localisation within or outside the cell where this abnormal phenomenon occurs. Similar information could be presented in a table (with the advantage that references can be included).

Minor remarks
- Not all abbreviations are needed. Especially when an abbreviation is not often used (and isn't a standard abreviation), it might be better to keep the full name to increase the readability.
- There are some formatting issues as some Greek letters are not formatted properly (e.g. a-synuclein)
- P. 13 + in K+ should be a superscript. In the rest of the text the full names are used of the different kations.
- Information on the clinical picture of FTD is lacking.
- p.14: FUS should be included in the list of causative genes for ALS.
- Table 2 'Can be obtained from adult donors'

Author Response Letter

Dear Professor Kastner and Professor Kao,

Re: PRONEU-D-20-00366

We are grateful for the reviewer's feedback and your important comments on our manuscript entitled **'Astrocytes and microglia in neurodegenerative diseases: lessons from human in vitro models'**, and for the opportunity to re-submit a revised version of our review article.

We were very pleased that the manuscript was positively reviewed. We very much appreciate all feedback received and believe that the comments have helped to enhance our manuscript. Taking each comment into careful consideration, we have produced the following point-by-point rebuttal, and we re-submit a revised manuscript which we hope will be suitable for publication in *Progress in Neurobiology*.

All changes to the manuscript are listed in detail in the point-by-point rebuttal below. Furthermore, all changes appear in the revised manuscript in blue for ease of reference.

Once again, we would like to thank the referees for all of their invaluable comments and feedback. We greatly appreciate their time in helping to improve our manuscript. We look forward to hearing back from you soon.

Yours Sincerely,

Rickie Patani

Comments from the Editors and Reviewers:

Reviewer #1: This review article by Franklin et al. nicely summarizes recent works using human in vitro models of astrocytes and microglia, and how they have been used to understand new aspects of glial involvement in neurodegenerative diseases. Overall, the manuscript is well written and provides a nice background on in vitro culture models, as well as the contribution of genetic variants to different neurodegenerative diseases. The tables and figures appropriately complement the text. Some minor comments are offered:

1) In Tables 2 and 4, karyotype abnormalities are listed as the only limitations for immortalized cell lines. Additional limitations of immortalized astrocytes and microglia could be listed here.

We thank the reviewer for this comment. We have amended Tables 2 and 4 to include further limitations of immortalized cell line derived astrocytes and microglia. To both Tables 2 and 4, we have added the following text:

"Abnormal proliferative state" and "Currently reliant on serum affecting reactivity"

2) In section 1, it is mentioned that inclusion of serum in astrocyte culture can have a profound influence on baseline reactivity. The matrix on which the cells are cultured can also contribute to the baseline properties of the cells, and it would be beneficial for the authors to provide some discussion on this point.

We thank the reviewer for highlighting this point. We have added the following text to address the influence of matrices on astrocyte phenotypes:

"In addition to the use of serum, matrix topography has been implicated in driving astrocyte reactivity in vitro (Puschmann et al., 2013; Watson et al., 2017). For example, astrocytes cultured in 3D collagen hydrogels and nanofibres are less likely to exhibit reactive phenotypes than those grown in 2D matrices (East et al., 2009; Tiryaki et al., 2015)."

3) The sentence "Furthermore, hiPSC derived astrocytes are only partially mature and are currently unable to capture age-related phenotypes" could be elaborated.

We have now elaborated on this point further to more clearly convey the advantages and disadvantages of the hiPSC model. We have added the following text:

"Furthermore, hiPSC derived astrocytes are only partially mature compared to adult astrocytes found in vivo and are currently unable to capture age-related phenotypes that have been described in astrocytes transdifferentiated from patient fibroblasts (Meyer et al., 2014)."

4) "Fluorescence activated cell sorting (FACS) can be used to purify cultures (Barbar et al., 2020), however, this is not necessary to obtain highly pure cultures" This statement could be further explained. What is considered sufficient purity for studies?

We thank the reviewer for this comment. We have amended this passage to make it clearer:

"Many protocols have successfully generated highly pure cultures of mature astrocytes (>90%) in the absence of fluorescence activated cell sorting (FACS), however FACS can be used as a tool to select for certain astrocyte-specific markers (Barbar et al., 2020)."

5) "However, several of these protocols produce macrophages which have not been validated as independent of the transcription factor MYB and vary in the yield of microglia obtained" Please explain the importance of MYB here.

We thank the reviewer for this comment and have now included further information to highlight the importance of MYB in generating CNS-specific microglia by editing the following text:

"However, several of these protocols produce macrophages which have not been validated as independent of the transcription factor MYB, a key determinant of the correct ontogeny to yolk sac-derived fetal macrophages (Schulz et al., 2012), and vary in the enrichment of microglia obtained (Buchrieser et al., 2017; Haenseler and Rajendran, 2019)."

6) "Astrocytes and microglia are able to undergo dramatic changes in gene expression in response to a wide array of stimuli in a process termed astrocyte reactivity". Consider subbing "astrocyte reactivity" with "reactive gliosis" which is inclusive of both astrocytes and microglia.

We thank the reviewer for this suggestion and have changed 'astrocyte reactivity' to 'reactive gliosis' accordingly.

7) The disease section mostly focuses on genetic variants that either cause or increase risk for the neurodegenerative diseases discussed. Additional commentary could be provided on how astrocytes/microglia can be/have been used to study non-genetic contributions.

We thank the reviewer for highlighting this important point. While studies that use such models to explore glial contributions to sporadic cases of neurodegenerative disease specifically are limited, we have made efforts to include this additional commentary.

To Section 3. Using human in vitro models to study astrocytes and microglia in neurodegeneration, we have added:

"With many genetic traits of neurodegenerative diseases occurring in glial cells, it is unsurprising that much focus has been on modelling astrocytes and microglia derived from patients with identified disease-linked mutations. iPSC-derived human in vitro models have provided a valuable insight into the pathogenic mechanisms that underlie neurodegenerative disease, but remain limited in the study of sporadic cases."

To Section 3.1.1 we have added:

"Such astrocyte pathology in AD has been recapitulated through various human in vitro iPSC models. iPSC-derived astrocytes from both familial and sporadic AD patients have been shown to exhibit marked disease phenotypes, including global morphological abnormalities and aberrant localisation of astrocytic markers (Jones et al., 2017).".

"APOE4-hiPSC derived astrocytes were also shown to be less efficient in Aβ uptake and clearance than APOE3-astrocytes, an effect that correlated with changes in lipid metabolism (Lin et al., 2018). Importantly, conversion of APOE4 to APOE3 in a sporadic AD line enhanced the ability of both hiPSC derived astrocytes and microglia to perform Aβ uptake."

"Increased reactive oxide species has also been observed in hiPSC microglia from sporadic AD patients, as well as increased phagocytosis in response to H_2O_2 treatment (Zhang et al., 2020)."

We also discuss some models of sporadic ALS in section 3.4.1:

"The neuroprotective capacity of hiPSC derived astrocytes to motor neurons has been recently demonstrated in a model of ALS using sporadic ALS (sALS) spinal cord extracts to seed aggregation of TDP-43 (Smethurst et al., 2020). Astrocyte conditioned media (ACM) from hiPSC derived astrocyte cultures reduced mislocalization and aggregation of cytoplasmic TDP-43 and improved motor neuron survival."

"Furthermore, astrocytes derived from transdifferentiated human fibroblasts from sporadic ALS (sALS) cases, mutant superoxide dismutase 1 (SOD1) and chromosome 9 open reading frame 72 (C9ORF72) cases also were toxic to co-cultured motor neurons (Meyer et al., 2014). sALS astrocytes transplanted into the spinal cords of immunodeficient mice induced motor neuron death and motor deficits (Qian et al., 2017)."

...

Reviewer #2: The review of Franklin et al. gives a nice overview of the potential involvement of astrocytes and microglia in different neurodegenerative disorders. In addition to an interesting overview of the differences between astrocytes and microglia and the different methods to obtain the different in vitro models, the review mainly concentrates on those phenotypes that were obtained using human in vitro models in different neurodegenerative disorders. The review is well structured, very well written and summarises a lot of information. It could be interesting for a very broad audience.

Major remarks

- The review is rather descriptive. It doesn't offer clear insights into what the human in vitro models exactly contributed to the different research fields. Apart from an overview of the obtained results, the review could have an important added value if a clear distinction could be made between occasional observations and important new insights and/or concepts which could potentially lead to completely new strategies to interfere with the different

neurodegenerative diseases. It might be too early to come to such a conclusion (see also next remark).

We thank the reviewer for this comment. Firstly, we have amended the text to more clearly highlight the contribution of human in vitro models to each research field. We have added the following text:

To section 2. Reactive transformation of hiPSC derived astrocytes and microglia:

"Human in vitro models have further demonstrated the cellular autonomy of astrocyte and microglial reactive processes, as highly purified populations of these cell types can be obtained that are naive to interactions with other cell types."

To section 3. Using human in vitro models to study astrocytes and microglia in neurodegeneration:

"Human in vitro hiPSC models of astrocytes and microglia are able to capture some disease phenotypes of these neurodegenerative diseases and demonstrate that a particular phenotype is cell autonomous. Furthermore, findings from human in vitro models of neurodegenerative diseases have begun to shed light on the primacy of pathological events."

Secondly, we have now highlighted important studies that may lead to new strategies in targeting neurodegenerative diseases, but agree that it is perhaps too early to come to this conclusion. We have also updated the manuscript to include new important studies that have been published since our review was submitted.

In section 2. Reactive transformation of hiPSC derived astrocytes and microglia, we more clearly highlight the importance of the recent comprehensive Barbar et al., 2020 study:

"The adoption of an A1 reactive state through stimulation with TNF-α, IL-1α and C1q has been recently recapitulated in an important comprehensive study using hiPSC derived astrocytes (Barbar et al., 2020). In this study, it was demonstrated that upon adoption of an A1 reactive state, astrocytes release proinflammatory factors whilst simultaneously losing homeostatic functions such as phagocytosis and glutamate uptake."

In section 3.1. Alzheimer's disease:

"APOE4-hiPSC derived astrocytes were also shown to be less efficient in Aβ uptake and clearance than APOE3-astrocytes, an effect that correlated with changes in lipid metabolism (Lin et al., 2018). Importantly, conversion of APOE4 to APOE3 in a sporadic AD line enhanced the ability of both hiPSC derived astrocytes and microglia to perform Aβ uptake."

"Recently, TREM2 knockout hiPSC microglia have been shown to elicit increased apoptotic cell death and impaired phagocytic ability (McQuade et al., 2020). In addition, TREM2 knockout

microglia transplanted into a mouse model of AD failed to effectively migrate and cluster around Aβ plaques."

"A 3D human tri-culture model of AD enabling the investigation of the important interactions between neurons, astrocytes and microglia has been recently developed (Park et al., 2018)... The development of this model has provided a unique opportunity to better

understand cellular interplay in the CNS as well as the influence of cell specific effects of AD-associated mutations and the potential interactions between different mutations occurring in astrocytes, microglia and neurons."

In section 3.2. Parkinson's disease:

"Another study, also using hiPSCs derived from PD LRRK2-positive patients, reported astrocyte- specific downregulation of genes associated with inhibition of microglial inflammatory responses and α-synuclein aggregate degradation (Booth et al., 2019), importantly implicating the loss of astrocyte neuroprotective capacity as a key player in the development of PD pathology."

"Recently, an elegant study uncovered a link between inflammation and neurodegeneration in PD using hiPSC-derived dopaminergic neurons and microglia carrying the LRRK2^{G2019S} mutation (Panagiotakopoulou et al., 2020). It was demonstrated that LRRK2^{G2019S} microglia display increased motility, phagocytic capacity and abnormal metabolic activity and immune responses to IFN-γ or LPS stimulation. Furthermore, LRRK2^{G2019S} but not control microglial conditioned media treated with LPS reduced neurite length of hiPSC dopaminergic neurons, directly implicating altered microglial immune responses in neuronal dysfunction in PD."

In section 3.4: Amyotrophic lateral sclerosis and frontotemporal dementia:

"TDP-43 mutant astrocytes have been shown to display TDP-43 mislocalisation (Serio et al., 2013). While these mutant astrocytes did not affect the survival of co-cultured neurons, an important observation was that mutant astrocytes can undergo cell death themselves."

"Furthermore, in an important study utilising astrocytes derived from transdifferentiated human fibroblasts from both sporadic and familial ALS cases including mutant superoxide dismutase 1 (SOD1) and chromosome 9 open reading frame 72 (C9ORF72), also were toxic to co-cultured motor neurons (Meyer et al., 2014).

- While the review mainly focuses on the phenotypes observed in the different in vitro models, information on the overall limitations of these in vitro models is lacking. At present, limitations are limited to the different model systems (Tables 2 and 4).

We thank the reviewer for detailing this oversight. We have included the following text in the conclusions to address this point:

"it is important to consider the limitations of any model system, as each have advantages and disadvantages depending on the scientific question to be addressed. Human in vitro models are more time-consuming and expensive to make than other in vitro models and cannot truly model the in vivo environment. It is also more difficult to model the complex multiple cell-cell interactions that exist in vivo. Furthermore, hiPSCs also represent a fetal maturational status which do not capture some age-related phenotypes that have particular importance to the study of neurodegenerative diseases (Patani et al., 2012; Ziff et al., 2018). Human in vitro models are nevertheless an incredibly important tool for investigating the primacy of pathological events and resolving cell autonomous pathological mechanisms."

- The quality and added value of the figures is limited. Text is too small and the added value is not so clear as it is mainly listing the mechanistic insights described in the text with lines pointing to the localisation within or outside the cell where this abnormal phenomenon occurs. Similar information could be presented in a table (with the advantage that references can be included).

We thank the reviewer for these comments. We have made efforts to improve the figure quality and have increased the size of the text. Since there are already a number of tables in the manuscript, we feel that although the information provided by the figures is in some respects limited, the figures do give the reader an easily digestible summary of prominent advances in each disease state.

Minor remarks
- Not all abbreviations are needed. Especially when an abbreviation is not often used (and isn't a standard abreviation), it might be better to keep the full name to increase the readability.

We agree and have reduced the number of abbreviations in the text to improve readability.

- There are some formatting issues as some Greek letters are not formatted properly (e.g. a-synuclein)

We thank the reviewer for pointing this out and have corrected these formatting issues.

- P. 13 + in K+ should be a superscript. In the rest of the text the full names are used of the different kations.

We have amended the text to be consistent throughout the manuscript.

- Information on the clinical picture of FTD is lacking.

We thank the reviewer for bringing this to our attention. We have now updated the manuscript with the following text: "Overlap of clinical, cellular and genetic aspects of disease with frontotemporal dementia (FTD), a disease characterised by progressive cognitive decline and behavioural abnormalities, has reframed ALS and FTD as part of a disease spectrum.

- p.14: FUS should be included in the list of causative genes for ALS.

We thank the reviewer for pointing out this oversight. We have added FUS to the list of ALS causative genes.

- Table 2 'Can be obtained from adult donors'

We have now amended the manuscript accordingly with this wording.
